# The Central Ohio Dental Association

**Published:** 1867-09

**Authors:** 


### Proceedings of Societies.

### THE CENTRAL OHIO DENTAL ASSOCIATION.

THE last meeting of this Association was held at Galion, Ohio, May 14th, 15th and 16th, 1867. A full attendance, interesting and instructive essays, able discussions, subjects of interest to the Dental profession, and a free interchange of views, with a kind, brotherly feeling prevailing, made this one of the most pleasant, as well as the most interesting meetings of the kind we ever had the pleasure of attending.

The following are the names of the officers elected for the ensuing year:

*President*—Dr. DeCamp, Mansfield, O.

*Vice-President*—Dr. C. M. Kelsey, Mt. Vernon, O.

*Secretary*—Dr. M. A. Spencer, Doylestown, O.

*Corresponding Secretary*—A. W. Maxwell, Galion, O.

*Treasurer*—Dr. H. M. Edson, Mt. Vernon, O.

Upon the close of a spirited discussion as to the conduct proper for a Dental practitioner, it was decided strictly unprofessional for a Dr. of Dental Surgery to travel from house to house, to solicit or perform operations, and we hope this may serve as a rebuke to some, (not members, however; they would not be allowed to do so) who are now doing this.

The following are the names of delegates elected to represent this Association in the American Dental Association, to convene in the city of Cincinnati, July 30th, and which is composed wholly of delegates from the different State societies:

Drs. E. Chidester, H. H. Harrison, A. W. Maxwell, W. E. Dunn, S. D. Stewart, J. M. Rhoads, G. W. Decamp, H. M. Edson, J. B. Beauman, M. A. Spencer, S. Wagoner.

Some able essays were read, for which the readers received votes of thanks, and which were ordered published in the DENTAL REGISTER, after being thoroughly discussed. A paper was read by Dr. G. W. DeCamp, entitled "Red Blood Globules," which called forth quite a discussion, joined in by Drs. Barber and Duff, physicians of Galion. "Dental Caries, its Causes and Arrest," was one of the regular subjects of discussion. It was ably discussed by many of the members, and no doubt all gained some valuable ideas.

Want of space will not allow further mention of this meeting, but suffice it to say, the Association finally adjourned, to meet in Akron, Nov. 12, 1867, and all returned to their offices and to their labor, refreshed and encouraged, and we hope, determined to study, as well as work, and strive to reach a higher position in their chosen profession.

M. A. SPENCER, *Secretary.*

